# Low-Level Visual Features of Window Views Contribute to Perceived Naturalness and Mental Health Outcomes

**DOI:** 10.3390/ijerph21050598

**Published:** 2024-05-06

**Authors:** Larissa Samaan, Leonie Klock, Sandra Weber, Mirjam Reidick, Leonie Ascone, Simone Kühn

**Affiliations:** 1 Clinic and Policlinic for Psychiatry and Psychotherapy, University Clinic Hamburg-Eppendorf, 20246 Hamburg, Germany; leonieklock@googlemail.com (L.K.); s.weber@uke.de (S.W.); m.reidick@uke.de (M.R.); l.ascone-michelis@uke.de (L.A.); s.kuehn@uke.de (S.K.); 2 Lise Meitner Group for Environmental Neuroscience, Max Planck Institute for Human Development, 14195 Berlin, Germany

**Keywords:** window view, image properties, low-level visual features, nature scenes, urban scenes, perceived naturalness, mental health, depression, anxiety, delay discounting

## Abstract

Previous studies have shown that natural window views are beneficial for mental health, but it is still unclear which specific features constitute a ‘natural’ window view. On the other hand, studies on image analysis found that *low-level visual features* (LLVFs) are associated with perceived naturalness, but mainly conducted experiments with brief stimulus presentations. In this study, research on the effects of window views on mental health was combined with the detailed analysis of LLVFs. Healthy adults rated window views from their home and sent in photographs of those views for analysis. Content validity of the ‘ecological’ view assessment was evaluated by checking correlations of LLVFs with window view ratings. Afterwards, it was explored which of the LLVFs best explained variance in perceived percentage of nature and man-made elements, and in ratings of view quality. Criterion validity was tested by investigating which variables were associated with negative affect and impulsive decision-making. The objective and subjective assessments of nature/sky in the view were aligned but objective brightness was unreliable. The perceived percentage of nature was significantly explained by green pixel ratio, while view quality was associated with fractals, saturation, sky pixel ratio and straight edge density. The higher subjective brightness of rooms was associated with a lower negative affect, whereas results for impulsive decision-making were inconsistent. The research highlights the validity to apply LLVFs analysis to ecological window views. For affect, subjective brightness seemed to be more relevant than LLVFs. For impulsive decision-making, performance context needs to be controlled in future studies.

## 1. Introduction

Previous research showed that nature exposure is beneficial for both physical and mental health (e.g., [1,2]) and that taking a walk in nature can lead to improved wellbeing (e.g., [3]) and cognition (e.g., [4]). These benefits have been shown for healthy (e.g., [1,3]) and for patient populations, including patients with depression or anxiety [5,6] as well as patients with physical illnesses such as cancer [7].

However, with increased urbanization, people tend to spend less time in natural environments. This comes with several health risks, as urban environments can induce stress (e.g., [8]) and potentially lead to negative effects on physical and mental health (e.g., [9]). Natural environments have repeatedly been reported to be more beneficial for mental health than urban environments (e.g., [1,2]). Similarly, natural elements in urban settings are associated with better mental health (e.g., [10,11]). Another aspect of urbanization is that more and more time is spent indoors. This is especially true for older populations or those with reduced mobility, but also a by-product of increased digitalization and jobs that require people to work inside. In addition, exceptional circumstances such as the COVID-19 pandemic, including measures such as isolation, can drastically increase the amount of time spent indoors. Also given that some studies predict the number of pandemics to increase in coming decades [12,13], it is highly important to investigate how the beneficial effects of nature on mental health can be applied to an indoor environment to protect mental health on a societal level. Furthermore, a more natural view—reflecting better ecology—is also of high importance given worldwide climate change and heat islands within cities, which have been shown to be a significant health hazard, including for mental health (e.g., heat-related increases in psychiatric emergencies, [14]). It has been proposed that to achieve a temperature reduction of 1 °C, a minimum tree coverage of 16% is generally required [15], with even higher levels of green having additional beneficial effects. Thus, increasing naturalness in window views could be a possible byproduct of enhancing urban green infrastructure, potentially also enhancing the wellbeing of indoor-dwelling citizens.

These factors make the view out of the window increasingly important. It also constitutes a form of connection to nature that is readily accessible to most people from inside their home. Previous research has demonstrated promising effects of window views on physical and mental health. In a pioneering study by Ulrich [16], patients recovered faster after surgery and took fewer potent analgesics when their hospital room window was facing trees compared to a brick wall. Similarly, Mascherek et al. [17] found that window views with a higher green pixel ratio and brightness in the room were associated with shorter hospital stays in patients treated for depression. With regard to private window views, people with a green window view from home had a significantly lower risk for anxiety and depression [18]. Further, Repke et al. [19] found that nature exposure and accessibility from home was significantly related to improved general wellbeing and health as well as lower depression, anxiety, and stress scores. Another study showed that participants whose homes had views with high amounts of diverse vegetation had significantly lower cortisol levels [20]. Relatedly, experimental studies indicated that window views including green space (compared to urban space) were associated with reductions in physiological stress [21,22]. Some studies directly investigated the effects of window views on mental health during the COVID-19 pandemic. For example, Pouso et al. [23] found that depression and anxiety symptoms during lockdown were lower for those with natural elements in their window view. Similarly, the existence of green window views from within the home was associated with increased levels of self-esteem, life satisfaction, and subjective happiness as well as with decreased levels of depression, anxiety, and loneliness during the COVID-19 lockdown [24].

Few studies directly investigated the association between window views and cognition, but Repke et al. [19] looked at the association between nature exposure from home (a self-report measure that included nature visible from one’s home, access to a yard, and green elements in the neighborhood) and mental health and cognition. While nature exposure from home was associated with better mental health (depression and anxiety scores), they found no significant associations with cognitive task performance (impulsive decision-making in a delay discounting task).

In summary, natural or ‘green’ window views seem to have beneficial effects on physiological and mental health, especially on depression, anxiety, and stress. Yet, most studies did not investigate which elements exactly constitute a ‘natural view’, but only differentiated between the absence or presence of nature or amount of green in a window view [18,23,24]. Further differentiating which elements make a view seem more natural (and thus, may lead to salutary effects on health) could help to incorporate these elements even in a predominantly urban environment and thereby structure environments in a way that enhances mental health.

Another line of research studies used image analysis to extract so-called low-level visual features (e.g., colors or edges) to analyze natural scenes [25,26,27,28,29]. It was found that natural and urban images differ significantly in their low-level visual features and that certain low-level visual characteristics are associated with perceived naturalness, preference, and positive valence. For example, higher perceived naturalness was positively associated with non-straight edge density and variability in saturation, but negatively associated with straight edge density and variability in hue [25,28,29]. However, some findings indicate that the recognition of features processed at a higher level (i.e., natural elements like trees or bushes) is crucial for the beneficial effects of nature on restoration [30,31] or that low-level visual features only partially mediate the relationship between naturalness and restorative qualities [26]. Thus, the calculation of low-level visual features should ideally be combined with subjective ratings of the same image/scene.

While studies on image analysis investigated the relationship between low-level visual features and mental health outcomes, they only performed this in experimental settings, often with very short exposure durations (confer [25,29]). This may not lead to ecologically valid results when investigating window views, since people are usually exposed to their real-life windows regularly, over longer time periods, and in a more indirect way than in a laboratory experiment.

The current study represents the (to our knowledge) first attempt to combine the ecological validity of studies investigating how regular exposure of window views at home relates to mental health (e.g., [18,23,24]) with the detailed observation of image analysis studies on low-level visual features (e.g., [25,28,29]) to investigate which low-level features vs. holistic perceptions (of ‘nature’ or ‘manmade’ components in the view) may ‘drive’ salutogenic (or conversely pathogenic) effects. We thus conducted an online study, in which participants sent in photographs of their window views at home. For all photographs, low-level visual features were calculated. In addition, participants reported the subjective perception of their window views (including the perceived percentage of sky, nature, and man-made elements in the view), answered questionnaires on mental health and performed cognitive tasks. 

The aim of the current study was two-fold. The first aim was to evaluate the content validity of extracting low-level visual features from ‘ecological’ window views. Therefore, first the validity for each low-level visual feature was assessed by testing associations of the extracted features from the images with subjective evaluations of the window views (% nature, % man-made, % sky, overall view quality) and by comparing results with the previous literature from image analysis. We hypothesized that objective measurements and subjective evaluations would be aligned and that we would be able to replicate most of the associations between specific low-level visual features and perceived naturalness found in previous studies [25,28,29]. Second, it was explored which of the low-level visual features best explained the variance in the perceived percentage of nature, perceived percentage of man-made elements, and overall view quality. We proposed that low-level visual features which have consistently shown positive (non-straight edge density, fractal dimension, standard deviation of saturation; [25,28,29]) or negative (straight edge density, standard deviation of hue; [25,28]) correlations with perceived naturalness to be important model predictors of the perceived percentage of nature or man-made elements, respectively, in the window view. Since nature is often equated with green [18,24], we expected the green pixel ratio to be of importance as well. Furthermore, we expected features that have previously been associated with preference (higher brightness, sky pixel ratio, mean and standard deviation of saturation as well as lower hue and straight edge density; [27,28,32]) to be important predictors of view quality ratings.

The second aim was to test the criterion validity of ‘ecological’ low-level visual window view features vs. subjective evaluations of window views, whereby associations with mental health and cognition were tested. This is the first study (to our knowledge) to test both low-level visual features and subjective perceptions of the window view concurrently and in competition to determine which variables dominate in explaining mental health and/or cognition. Therefore, we used an exploratory approach and had no predictions about which features would prevail. The aim was to identify important window view variables to help future research generate more targeted hypotheses. Ultimately, identifying these features could help create more health-promoting window views, e.g., by informing urban planning and design choices based on objective low-level visual features and/or subjective perceptions that are relevant to mental health.

We focused on subclinical depressive symptoms and anxiousness as mental health parameters, since these were investigated in previous studies on window views [18,23]. Furthermore, given the high prevalence of depression and anxiety worldwide [33], especially in urban environments [34], finding ways to improve these mental health outcomes is a globally relevant public health concern. For cognition, we assessed impulsive decision-making in a delay discounting task, whereby previous studies showed mixed findings with regard to nature and impulsive decision-making [35,36] and the specific association between window views and impulsive decision-making remains therefore unclear (see [19]). Impulsive decision-making in delay discounting tasks is related to health behaviors like smoking, nutrition, and exercise frequency (e.g., [37]); therefore, increasing delay discounting could help to improve various health outcomes in the population.

## 2. Materials and Methods

### 2.1. Participants

A total of 110 participants took part in the study. The mean age was 26.8 years (*SD* = 7.99, age range 18–62 years), with 81 participants identifying as female and one participant identifying as diverse. Most participants were living in a city with a population of over 100,000 (*n* = 96) and 90% had a higher education entrance qualification (*n* = 99). More detailed sociodemographic information can be found in Table 1. The study was approved by the local psychological ethics committee of the center for psychosocial medicine at Universitätsklinikum (University Clinic) Hamburg-Eppendorf (approval code LPEK-0128) and preregistered (https://aspredicted.org/ke2wu.pdf, accessed on 14 March 2024). Participants were recruited online via German job platforms (‘Stellenwerk’ and eBay ‘Kleinanzeigen’) between May and June of 2020, right after the first COVID-19 lockdown in Germany. They were informed that the study’s goal was to assess associations of neighborhood and window view features with wellbeing and cognition. The inclusion criteria were being 18–75 years of age, a current residency (since the beginning of the restrictions during the COVID-19 pandemic) in the same apartment where they spent most of their time in the last 6 months before the restrictions, and the willingness to participate in an online study lasting approximately 60–90 min and to subsequently submit photos of their own windows via the ‘MyPhotoApp’, an app specifically developed for creating and securely uploading window view photos. The exclusion criteria were lifetime neurological or psychiatric disorders. All inclusion and exclusion criteria were clearly stated in the recruitment information participants received. After both completing the online study and sending in photos of their window views, participants received EUR 15.00 for their participation.

### 2.2. Study Design and Procedure

Before beginning the study, participants gave their informed consent. They then answered demographic questions as well as questions about their neighborhood concerning upbringing, the current neighborhood, window views, the quality of living in their current home, as well as questions regarding the isolation during the first COVID-19 lockdown (e.g., how much time they spent indoors compared to prior to the lockdown). Afterwards, they answered nine questionnaires and performed four cognitive tasks in the following order: forward and backward digit span tasks [38], the connectedness to nature scale [39], the WHOQOL-BREF quality of life assessment [40], the WHO-5 wellbeing index [41], the wellbeing scale by Ryff and Keyes [42], the satisfaction with life scale [43], the digit symbol substitution test [44], the positive and negative affect schedule (PANAS; [45]), the state-trait anxiety-depression inventory (STADI; [46]), a delay discounting task [47], the perceived stress scale [48], a Necker cube task [49], and a self-control scale [50]. The online study was conducted in German and took 60 to 90 minutes to be completed.

The current study focuses on demographic data, window data, the STADI [46] and the delay discounting task [47], which are described in detail below. As mentioned in the introduction, we focused on subclinical depressive symptoms and anxiousness because previous studies show the beneficial effects of ‘natural’ window views on these outcomes, and we wanted to examine which specific features of a window view could explain these effects. Further, since previous studies showed inconsistent findings regarding impulsive decision-making, we wanted to specifically investigate the association between window view features and decisions in a delay discounting task. 

After completing the online study, participants received a personalized link to a custom-made app on their phone with which they could photograph their window views (‘MyPhotoApp’). Participants were instructed in detail on how to take photographs of their window views: photos should be taken during a bright time of the day (morning or noon), objects that are permanently in front of the window (e.g., plants on window sills or curtains) should not be removed, milk glass or any other windows should be photographed while being closed, and no people should be in the pictures. Participants should stand straight in front of the window and place their camera at eye level while taking the photos. Importantly, window frames or the limits of each window should be visible in the images. The exact instructions can be found in Appendix A.

### 2.3. Materials

The study was programmed using Inquisit 6 [51] and distributed via the German job platforms ‘Stellenwerk’ and eBay ‘Kleinanzeigen’. 

#### 2.3.1. Questions on Window Views and Living Situation

Participants provided subjective ratings of their window views, which were then used to assess the content validity of the low-level visual features. Amongst other questions, participants were asked to estimate the percentage of nature (‘In your estimation, what percentage of what you see from the windows of your home is nature (without sky), e.g., trees, plants, meadow, water, etc.?’), man-made elements (‘In your estimation, what percentage of what you see from the windows of your home is man-made, e.g., road, building, etc.?’), and sky (‘In your estimation, what percentage of what you see from the windows of your home is sky?’) in their window views (averaged across all windows at their home). The sum of these estimates had to add up to 100%. 

The participants also rated the overall quality of the view (‘How would you rate the quality of your apartment in terms of the following features? Window views’), the visibility of greenery through their windows (‘Visibility of greenery (trees, meadows, etc.) from the windows’) as well as the average brightness of the rooms in their home (‘Brightness of the rooms during the day’) on a scale from zero (‘very bad’) to 100 (‘very good’). They also indicated the number of rooms out of which they were able to see vegetation (‘From how many rooms can you see vegetation? (e.g., trees, shrubs, lawns, fields)’). Finally, participants indicated how many hours per day they spent inside/outside their homes on average before and during the lockdown (‘How much time did you spend in your home every day before the current COVID-19 restrictions (hours per day)?’; ‘How much time do you spend outside your home since the current COVID-19 restrictions (hours per day)?’).

#### 2.3.2. Predicting Perceived Naturalness, Perceived Man-Made Elements, and View Quality with Low-Level Visual Features

To evaluate the content validity of the low-level visual feature assessment method for ‘ecological’ window views, we calculated low-level visual features for each window image as described below. LLVFs were then intercorrelated to check whether we would find patterns as to-be-expected (e.g., a positive association between green pixel ratio and entropy as well as fractals, since all three parameters are related to higher naturalness). In addition, in a stepwise regression approach, we were aiming to explore which of the LLVFs best explained variance in perceived naturalness, man-made elements, and overall window view quality as well as how much variance of these outcomes could be explained using LLVFs (for details see Data Analysis subsection).

*Image Analysis to Obtain Low-Level Visual Features.* Each window image was preprocessed as follows: First, window frames and other parts outside of the window view were removed and the image was straightened out, so that only the window view without the window frame remained in the image. Objects in front of the window view (e.g., plants or curtains) were deliberately left in the image, since ecological validity was explicitly desired in evaluating the low-level visual features and participants were instructed to leave everything as usual when taking the picture. For the calculation of the sky pixel ratio, a second image set was created. Here, everything that was not sky was manually blackened. Image preprocessing was performed in GIMP (version 2.8.22, The GIMP Development Team, https://www.gimp.org (accessed on 14 March 2024)) and Adobe Photoshop (version CS5, Adobe Systems Software Ireland Limited, Dublin, Republic of Ireland). We then calculated the average values across all window images of each participant, which resulted in one value for each low-level visual feature per participant. These average values were used for further correlation and regression analyses.

The color properties of the standard HSV model (Hue, Saturation, and Value) as well as the spatial properties entropy and different types of edge densities were calculated using a MATLAB script [52] adapted from a script by Berman et al. [25] which utilizes the MATLAB Image processing toolbox functions (MATLAB and Image Processing Toolbox Release 2012b, The MathWorks, Inc., Natick, MA, USA). A detailed description of how entropy and edge densities were extracted can be found in Berman et al. [25]. In addition, green pixel ratio, blue pixel ratio, and sky pixel ratio for each image were calculated using Python utilizing the package NumPy [53], OpenCV [54], and the glob module of the Standard Python Library (confer [17]). Fractal dimension was calculated in binary image versions using the box-counting method [55,56] from the FracLac add-on to the ImageJ software [57,58]. The following section describes each of the objective low-level visual features and how they were calculated in the current study.

*Exemplary Analysis of Window Views with Low-Level Visual Features.* Table 2 shows two exemplary window views and their respective low-level visual features. In order to protect the privacy of our study participants, we chose window photographs of one of our offices at the University Medical Center Hamburg-Eppendorf. We derived 14 objective low-level visual features, which are described in detail below.

We calculated color properties of the standard HSV model (Hue, Saturation, and Value; confer [25]) as well as the green pixel ratio, blue pixel ratio, and sky pixel ratio for each image.

**Figure 1:**
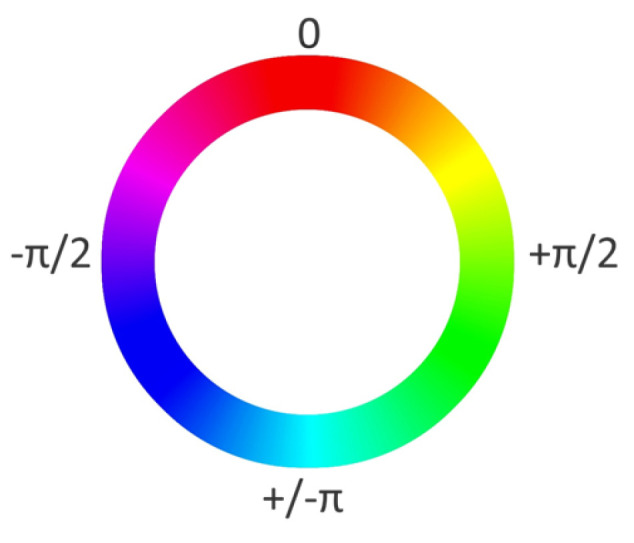
Color circle.

1. Hue describes the average color dimension of an image [29]. This average can be described as a specific position on a color circle (see Figure 1). In the current study, hue values range from −π to +π, where the hue for cyan is set to −π and +π and the hue for red is set to zero. Due to the circular nature of the hue, the lowest value equals the highest value. The exemplary window images have an average hue of 1.74 and 1.54, respectively, indicating that both images depict a lot of green color.
2. The standard deviation (*SD*) of hue describes the degree of diversity in an image’s hue [25]. Window 1 has a larger *SD* of hue than Window 2, indicating a higher diversity of color in Window 1. In previous studies, a lower *SD* of hue was associated with higher perceived naturalness [25,28,29].
3. Saturation describes the ratio of the dominant color wavelength compared to all other color wavelengths in a pixel [25]. We calculated the average saturation across all pixels for each window image. In the current study, saturation values ranged from zero to one, with a higher value indicating a higher saturation. Both exemplary window images show rather low saturation values.
4. The *SD* of saturation depicts the variability of saturation within an image. The exemplary window images have almost identical *SD*s of saturation. Previous studies showed a positive association between the *SD* of saturation and perceived naturalness [25,28,29].
5. The brightness (also: luminance or value) of an image is the overall darkness-to-lightness of a pixel depending on the pixel’s brightness [25]. Brightness values range from zero to one. Window 2 has a higher brightness than Window 1, but both images have low to medium brightness.
6. The *SD* of brightness describes the variability of brightness within an image and is similar to the contrast of an image [29]. Again, the exemplary window images have very similar and rather low *SD*s of brightness.
7. The green pixel ratio describes the ratio of green pixels in an image to all pixels in an image. Values can range from zero to one. Window 1 has a higher green pixel ratio than Window 2. Notably, the trees in the exemplary windows have darker parts of leaves that appear black or brown in a photograph, although a person looking out of the window might still perceive them as green.
8. Similarly, the blue pixel ratio is the ratio of blue pixels in an image to all pixels in an image. Both exemplary window images have fairly low blue pixel ratios.
9. The sky pixel ratio describes the ratio of the sum of blue and grey/white pixels in an image to all pixels in an image. Importantly, image aspects that are not sky had to be painted black prior to the extraction of sky pixel ratio, so that other blue and grey elements in the images are not mistaken for sky. Both exemplary window images show low to medium sky pixel ratios, with Window 2 having a slightly higher amount of visible sky.

We also calculated spatial properties for each window view image, namely, entropy and edge density measures (confer [25]) as well as fractal dimension [29,59].

10. Entropy is a measure of the average information content of an image. It is higher in complex images with randomly arranged elements, hence it is often used as an indicator of randomness in an image [25,29]. The average values of entropy can range from zero to eight, but most natural scenes have a value between seven and eight. Window 1 has a higher entropy than Window 2, indicating more randomness.
11. We measured three different forms of edge density: The overall edge density of an image as well as its sub-categories straight edge density and non-straight edge density. In general, the (overall) edge density of an image can be described as the quantification of well-defined edges, curves, and lines in an image. It is calculated by dividing the pixels on edges by all pixels of an image [29]. Edge density values usually range from zero to 0.5, so the exemplary windows show low to medium edge densities. In previous research, overall edge density was positively associated with perceived naturalness [25,28,29].
12. Straight edge density is a sub-category of edge density that describes the ratio of pixels on straight edges (i.e., horizontal, vertical, or oblique lines) to all pixels of an image [25]. A higher straight edge density has been found to be associated with lower perceived naturalness [25,28,29].
13. In contrast, non-straight edge density is calculated with the ratio of pixels on non-straight edges (i.e., curved, or fragmented lines) to all pixels of an image [25]. The exemplary window images both depict more non-straight than straight edges. A higher non-straight edge density was associated with a higher perceived naturalness in previous studies [25,28,29].
14. Finally, we calculated the fractal dimension for each image. Fractals are repetitive patterns, in which a larger object is composed of geometrically identical, smaller objects [60]. Fractal dimension is often used as a measure of complexity [29]. In this paper, fractal dimension values range from one to two, which means that both exemplary windows have rather high fractal dimension values.

#### 2.3.3. Predicting Mental Health Outcomes with Window View Parameters

To test the criterion validity of ‘ecological’ low-level visual window view features vs. subjective evaluation of window views, we examined the relationship of these features and evaluations concurrently in an exploratory stepwise regression approach with the outcomes negative affect (anxiety and depression) and delay discounting (impulsive decision-making; for further details see Data Analysis subsection).

*State-Trait Anxiety-Depression Inventory (STADI).* The STADI [46] consists of 40 items that measure the current (STADI state, 20 items) and general anxiety and depression (STADI trait, 20 items) level of a person with four subscales. The current study focuses on the state version and participants were asked to relate the statements to their ‘current state’. State anxiety (e.g., ‘My heart is beating fast’) and state depression (e.g., ‘I feel like crying’) are further divided into emotionality and worry items (anxiety), and euthymia and dysthymia (depression) items. Euthymia items are reversely scored. Each item has four response options, ranging from 1 (not at all) to 4 (very much so). Based on the manual [46], the subscales can be combined in different ways. For our purposes, we added up the scores of the state depression and the state anxiety subscales, leading to a composite score for state negative affect. A higher sum score reflects a higher state negative affect. Note that while the STADI has been validated for non-clinical and clinical populations, it is often used in healthy populations and the current study also refers to a non-clinical sample, which means that our results do not reflect effects on mental illness, but rather on transient depressive states and anxiousness in healthy adults.

*Delay Discounting Task.* In the delay discounting task ([47], see also [61]), participants were presented with the choice of either receiving EUR 50 immediately or EUR 100 at a delayed point in time. These delays varied between blocks and could be six hours, one day, one week, one month, three months, one year or five years. The order of blocks was randomized, and participants performed six trials per block/delay [47]. If a participant chooses the immediate reward, the amount of the reward is reduced by 50% in the next trial (i.e., EUR 25 now or EUR 100 at a later point in time). If, on the other hand, a participant chooses the delayed reward, the amount of the immediate reward is increased by 50% in the next trial (i.e., EUR 75 now or EUR 100 at a later point in time). In subsequent trials, the amount is adjusted, respectively, with increasingly smaller changes. The goal is to find the indifference point of a participant which refers to ‘a point at which the present value of the delayed alternative is equal to that of the immediate alternative’ (p. 2, [62]). The seven indifference points (one for each delay) can be used to calculate the area under the curve (AUC) for a participant (see [63]), whereby a larger AUC represents less discounting by delay, i.e., less impulsive decision-making or higher impulse control [64].

#### 2.3.4. Data Analysis

To evaluate content validity, we first calculated univariate Spearman correlations between all low-level visual features and subjective window view ratings. This first step was conducted to validate the extracted features from the window view in terms of their intercorrelations, their relationship with participant perceptions, and their consistency with findings from the previous literature. 

Next, automated stepwise linear regression analyses were performed to see how much of the overall variance of subjective participant ratings of perceived percentage of nature and man-made elements in the window view as well as indicated view quality could be explained by objective low-level visual features. This atheoretical approach—in which all features were tested simultaneously (see Table 3)—was adopted because to our knowledge, no previous study had investigated low-level visual features of window views, and the research question was therefore highly exploratory. We used a bidirectional stepwise approach since it removes some of the issues associated with forward stepwise regression [65]. At each step, additional variables were included based on the AIC (Akaike information criterion), and the model with the lowest AIC was selected as the final model. The robustness of these identified ‘explanatory’ low-level visual features was checked by adding control variables, including sociodemographic confounders, in a second stepwise regression. More specifically, the low-level visual features that were previously identified were entered in a fixed block, and thereafter a second block of control variables, which were selected in the same stepwise manner, was added (see Table 4).

Further, to assess criterion validity, we examined whether and to which degree both subjective ratings of the views and low-level visual features were significant predictors in relation to the outcomes of interest: negative affect and impulsive decision-making (delay discounting). Therefore, we again used hierarchical (first block: control variables, second block: low-level visual features and subjective ratings of window views) bidirectional stepwise linear regression. All variables in the first block were entered by ‘forced entry’ (since they constituted theoretically relevant confounders); for the second block we used stepwise selection of variables. For details, see Table 5. Again, an atheoretical approach was adopted because to our knowledge, there is no consistent previous research on how and which specific low-level visual features and subjective ratings of window views affect mental health and cognition. Again, variables were chosen based on the AIC at each step and the model with the lowest AIC was selected as the final model. As may become apparent, the order of blocks (sociodemographic confounders first, window view parameters second) was interchanged compared to the previous analyses (see Table 4). This was undertaken because sociodemographic confounders (e.g., sex) were considered to be more proximal to mental health than window view parameters, whereas we suspected window view ratings to be better explained by low-level visual features than by sociodemographic confounders. 

We excluded the one participant with diverse sex from all correlation and regression analysis because a level with one person could distort results. Furthermore, participants with missing data on relevant outcomes were excluded from the respective analyses; the adjusted sample sizes are reported in the results section. The correlation and regression analyses were conducted in RStudio [66] with RStudio version 2023.6.1.524 (Posit Software, PBC, Boston, MA, USA) and R version 4.2.3 (2023-03-15 ucrt; R Foundation for Statistical Computing, Vienna, Austria). For the correlation analyses, we used the corrplot package (v0.92; [67]) and the rcorr function of the Hmisc package [68]. Model assumptions were visually examined with the plot_model function of the sjPlot package [69]. We thereafter used the stepwise function of the StepReg package [70] for stepwise regression and the step function of the stats package [71] for hierarchical stepwise regression. The standardized beta coefficients were calculated using the lm.beta function of the QuantPsyc package [72]. The results are considered statistically significant in the case of *p* ≤ 0.05. 

## 3. Results

**Figure 2:**
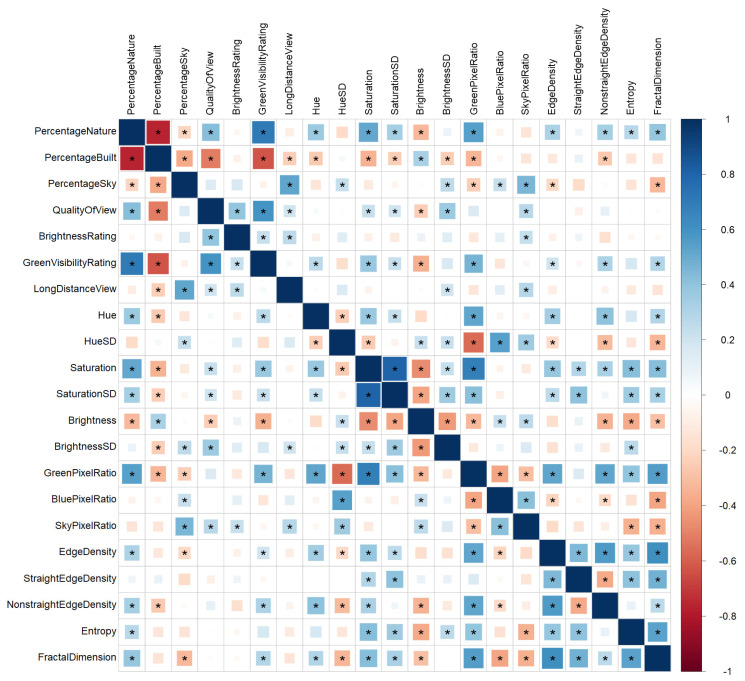
Spearman correlations between low-level visual features and subjective window ratings. PercentageNature = perceived percentage of nature in the window view; PercentageBuilt = perceived percentage of man-made elements in the window view; PercentageSky = perceived percentage of sky in the window view; QualityOfView = rating of overall view quality; BrightnessRating = rating of brightness in the room; GreenVisbilityRating = rating of visibility of green in the window view; LongDistanceView = estimation how far one can see out of the windows. Low-level visual feature values were averaged across all window views of each participant. *SD* = Standard Deviation. Larger squares represent larger correlations. * *p* < 0.05.

Figure 2 depicts a correlation matrix of all subjective window view ratings as well as the objective low-level visual features. The following sections present these correlational results in detail.

### 3.1. Content Validity of Extracting Low-Level Visual Features from ‘Ecological’ Window Views

#### 3.1.1. Correlations between Subjective Ratings and Objective Low-Level Visual Features

*Subjective Nature/Green Perception vs. Green Pixel Ratio from Window Views*. In the survey, participants estimated the percentage of nature (compared to the percentage of sky and man-made elements) in their window views and rated the visibility of green. In addition to these subjective ratings, we calculated the (objective) green pixel ratio for each image. Spearman correlations revealed significant positive correlations between the green pixel ratio and perceived percentage of nature (ρ = 0.55, *p* < 0.05) and between the green pixel ratio and ratings of green visibility (ρ = 0.47, *p* < 0.05). Correspondingly, we also found a significant negative relationship between the green pixel ratio and the perceived percentage of man-made elements (ρ = −0.34, *p* < 0.05). These results confirm that the objective and subjective estimates of nature and green are aligned. 

*Subjective Percentage of Sky vs. Sky Pixel Ratio from Window Views.* The perceived percentage of sky in the window view was significantly positively correlated with the objective sky pixel ratio (ρ = 0.45, *p* < 0.05), indicating that the subjective estimates and objective assessments of sky are similar.

*Subjective Brightness at Home vs. Objective Brightness from Window Views.* There was no correlation between the subjective ratings of brightness and the objective assessment of brightness in the window view pictures (ρ = 0.09, *p* = 0.37). There are multiple possible explanations for this finding. First, while the objective assessment reflects the brightness within the window view (averaged across all windows of a participant), the subjective ratings refer to the average brightness in the rooms of an apartment. These two do not necessarily correlate positively, since, e.g., a small bright window might not be large enough to light up an entire room. Second, brightness is a volatile phenomenon (ephemeral feature) that changes throughout the day, as well as by season and other factors. It could therefore be that the brightness in the window photographs does not reflect the brightness level that participants generally perceive on a daily basis. Finally, sanity checks of all window photographs that were sent in by participants revealed that the brightness of a window image varied highly depending on camera settings, leading to large differences in the contrasts between the window view and curtains, window frames, and other objects. All in all, it seems like the objective assessment of the brightness of an image as in the ecological approach presented herein is not a reliable way to measure the brightness of a window view in a private residence. Therefore, we excluded objective brightness (average and *SD*) from further analyses.

#### 3.1.2. Correlations of Objective Low-Level Visual Features with Perceived Naturalness and Quality Ratings

To further validate the ecological window view approach, we aimed at replicating previous studies that investigated how low-level visual features of images relate to perceived naturalness and preference [25,27,29]. Thus, we calculated univariate associations between low-level visual features and ratings of view quality as well as perceived percentage of nature or man-made elements in the window view and compared our findings to those of previous studies. 

The rating of the view quality was significantly positively correlated with the perceived percentage of nature (ρ = 0.42, *p* < 0.05) and significantly negatively correlated with the perceived percentage of man-made elements (ρ = −0.52, *p* < 0.05). Concerning low-level visual features, the overall pattern of findings suggested validity, since the *SD* of saturation, overall edge density, non-straight edge density and fractal dimension were significantly and positively associated with perceived naturalness (all ρ > 0.31 and all *p* < 0.05), as similarly found in previous studies with experimental images [25,28,29]. Like in these previous studies, we found a negative correlation between the *SD* of hue and naturalness, however, this effect did not reach significance (ρ = −0.18, *p* = 0.06). We were not able to replicate the negative association between straight edge density and naturalness (ρ = 0.07, *p* = 0.48) that was reported in previous studies [25,28,29]. For all other low-level visual features (hue, saturation, brightness, and entropy), associations with naturalness and preference were not consistent across previous studies (see [25,27,28,29,73]), thus the results in the present study are in line with some but not all of the existing literature. Inconsistent findings across studies could be due to varying effects of certain low-level visual features on perceived naturalness. Alternatively, some of our contradictory findings could be due to window views being perceived differently than other nature/urban scenes, or other specific features of the images used in our study. 

Previous studies did not directly measure green, blue, and sky pixel ratio. Our results suggest that, unsurprisingly, green pixel ratio was positively associated with the perceived percentage of nature (ρ = 0.55, *p* < 0.05) and negatively associated with the perceived percentage of man-made elements (ρ = −0.34, *p* < 0.05). Both the green pixel ratio and sky pixel ratio were positively related to view quality, but only the correlation with the sky pixel ratio reached significance (green pixel ratio with overall view quality: ρ = 0.15, *p* = 0.11; sky pixel ratio with overall view quality: ρ = 0.27, *p* < 0.05). The blue pixel ratio showed no significant correlations with view quality (ρ = 0.01, *p* = 0.96) nor with perceived percentage of nature (ρ = −0.07, *p* = 0.50) or man-made elements (ρ = −0.05, *p* = 0.63). A detailed feature-by-feature report of validity check results can be found in Appendix B.

### 3.2. Predicting Perceived Naturalness, Perceived Man-Made Elements, and View Quality with Low-Level Visual Features

#### 3.2.1. Low-Level Visual Features Explaining Perceived Percentage of Nature in Window Views

The first stepwise linear regression analysis resulted in the model shown in Table 6, including the green pixel ratio and saturation as predictors for the perceived percentage of nature in the windows (AIC_model 1_ = 629.24 compared to AIC_intercept only model_ = 766.64), whereby overall about 23% of the variance in the perceived percentage of nature was explained, *F*(2, 106) = 17.05, *p* < 0.001. Both predictors were positively associated with the perceived percentage of nature. The green pixel ratio turned out to be a significant predictor (*p* < 0.01) whereas the effect of saturation did not reach significance (*p* = 0.11), however, the model fit was better when including saturation and the green pixel ratio (AIC = 740.24), compared to including the green pixel ratio alone (AIC = 740.95). Adding a second block with control variables using hierarchical stepwise regression resulted in the same model, i.e., adding any of the control variables would not improve the model fit. 

#### 3.2.2. Low-Level Visual Features Explaining Perceived Percentage of Man-Made Elements

The final model (see Table 7) included the green pixel ratio, sky pixel ratio and *SD* of hue as predictors for the perceived percentage of man-made elements (AIC_model 1_ = 759.61 compared to AIC_intercept only model_ = 775.86), whereby overall about 16% of the variance in the perceived percentage of man-made elements was explained, *F*(3, 105) = 7.92, *p* < 0.001. All predictors were significant (*p* < 0.05) in a negative direction, i.e., higher values relating to a lower perceived percentage of man-made elements in the window view from the home. Again, adding control variables in a second block resulted in the same final model, i.e., adding any control variable would not improve the overall model fit. 

#### 3.2.3. Low-Level Visual Features Explaining Ratings of View Quality

Model 1 included saturation, the sky pixel ratio, fractal dimension and straight edge density as predictors for the ratings of view quality (AIC_model 1_ = 807.63 compared to AIC_intercept only model_ = 816.64), whereby about 11% of the variance in ratings of view quality was explained, *F*(4, 104) = 4.39, *p* = 0.003. Saturation, the sky pixel ratio and fractal dimension were significantly (*p* < 0.05) and positively associated with a higher view quality rating, while straight edge density was non-significantly negatively associated with the view quality rating; however, it was automatically included in model 1 because it improved the AIC. Adding a second block with control variables in the same stepwise manner resulted in model 2, with one other variable being automatically included: the number of images that were sent in by participants was significantly positively related to the view quality rating of the window views. Including this variable in the final model (i.e., model 2) improved the AIC (AIC_model 2_ = 693.83 compared to AIC_model 1_ = 696.63). In model 2, about 14% of the variance in ratings of view quality was explained, *F*(5, 103) = 4.569, *p* < 0.001. Both models are shown in Table 8.

### 3.3. Predicting Mental Health Outcomes with Window View Parameters

Spearman correlations between all variables (including mental health outcomes) are reported in Appendix C.

#### 3.3.1. State Negative Affect

For detailed results, see Table 9. The hierarchical stepwise linear regression analysis resulted in a model that included all control variables (due to forced entry in a first hierarchical block; however, none of them were significant) plus subjective brightness, entropy, and straight edge density that were automatically included based on the stepwise approach in the second block (AIC_final model_ = 485.68 compared to AIC_control variables only_ = 499.07). However, only the subjective rating of brightness in the rooms was a significant predictor (*p* < 0.01), with a higher rating of brightness being associated with lower state negative affect. In the final model, 15.5% of the variance in state negative affect was explained, *F*(10, 96) = 2.94, *p* = 0.003.

#### 3.3.2. Delay Discounting

In addition to the control variables (included based on forced entry in a first block), the final model included saturation, overall edge density, fractal dimension, the rating of green visibility, and the perceived percentage of nature as predictors for impulsive decision-making in a delay discounting task (AIC_final model_ = 686.30 compared to AIC_control variables only_ = 692.16; see Table 10). Among the control variables, the time that participants spent at home on average was significantly positively associated with less discounting by delay, i.e., less impulsive decision-making. A higher color saturation in the window view was also associated with less impulsive decision-making. On the other hand, overall edge density as well as the perceived percentage of nature in the window view were significantly positively associated with more impulsive decision-making. In the final model, about 12% of the variance in delay discounting was explained, *F*(12, 95) = 2.24, *p* = 0.02.

## 4. Discussion

The current online study aimed to combine the ecological validity of investigating the effects of real window views of private residencies [18,23,24] with the detailed approach of image analysis studies [25,29] and to concurrently examine objective low-level visual features and subjective ratings of window views in their association with mental health and cognition. First, we examined correlations between objective low-level visual features and subjective perceptions of window views to replicate previous findings on the relation between low-level visual features and perceived naturalness (e.g., [25]). Next, we used an atheoretical approach applying stepwise linear regression analysis to identify objective predictors for the perceived percentage of naturalness and man-made elements, and for the overall view quality of the windows. Finally, we investigated how low-level visual features and subjective ratings of windows can explain the mental health outcomes, state negative affect and impulsive decision-making (delay discounting), again using stepwise linear regression while including and controlling for socioeconomic confounders in the analyses.

### 4.1. Content Validity of Extracting Low-Level Visual Features from ‘Ecological’ Window Views

A detailed report and discussion of the results concerning content validity can be found in Appendix B. Overall, the intercorrelation pattern between low-level visual features as well as the correlative pattern between low-level visual features and perceived naturalness (and sky) suggests that the ‘naturalistic approach to window views’ in the present study was valid, with the exception of objective brightness (average and *SD*), which was unreliable due to different lighting conditions and camera settings and therefore was excluded from further analyses.

### 4.2. Predicting Perceived Naturalness, Perceived Man-Made Element, and View Quality with Low-Level Visual Features

In order to identify the most important predictors for perceived percentages of nature and man-made elements as well as for the ratings of view quality, we used stepwise linear regression. A higher green pixel ratio was the most important (and also only significant) variable related to a higher perceived percentage of nature and was also significantly related to a lower perceived percentage of man-made elements. This confirms that previous studies were headed in the right direction by asking about the presence vs. absence of nature or green in the window view (confer [18,23,24]). However, there may be a dose-dependent relationship between the amount of nature in the view and mental health outcomes. Thus, even when refraining from the detailed analyses of low-level visual features, studies should classify the amount of green in the view, instead of only the presence or absence of green, as well as provide more detailed descriptions of the type of nature since the quality of green also plays an important role (for a review on this topic see [74]). Stepwise exploratory regression revealed that model fit was better when saturation was included as an additional predictor for perceived naturalness, albeit AIC was only slightly smaller, and the effect of saturation did not reach significance. While the significance of green pixel ratio in predicting perceived naturalness is in line with our hypothesis, we expected other low-level visual features (e.g., non-straight edge density or saturation) to be of similar importance, which did not prove to be the case.

Concerning man-made elements, other significant predictors besides the green pixel ratio were the sky pixel ratio and the *SD* of hue. A higher sky pixel ratio was associated with a lower perceived percentage of man-made elements. This could be simply due to the fact that a higher proportion of visible sky in the window view indicates the absence of highly dense or high buildings. While most studies on nature and mental health operationalize nature as green or blue spaces, Sztuka et al. [32] investigated whether sky is perceived as nature as well. They found a small but significant positive association between the amount of sky and the naturalness ratings of images. Surprisingly, a higher *SD* hue was also associated with a lower perceived percentage of man-made elements in our study. This contradicts previous studies (and our hypothesis based on them) which found that natural environments have lower *SD* hues than built environments [25,28,29]. Similarly, in our study, the univariate correlation between the *SD* of hue and perceived percentage of nature was negative, albeit this correlation was not significant. However, the linear model calculates the effect of *SD* hue given that the other predictors in the model are held constant, i.e., if the effects of the sky pixel ratio and green pixel ratio are held constant, the *SD* of hue has a negative effect on the perceived percentage of man-made elements. It could therefore be that natural environments have previously been associated with a lower *SD* of hue because most natural environments are mainly green or blue and therefore have less color variation than built environments, which can include all types of colors. But if the amount of green and blue (represented by the amount of sky) is held constant, the *SD* of hue could actually have the opposite effect.

Subjective window view quality was best explained by higher sky pixel ratio, saturation, and fractals, as well as by lower straight edge density. These findings are in line with our hypotheses based on previous research on preference [27,28,32]. This highlights the preference for natural window views, given that saturation and fractals are associated with perceived naturalness and straight edge density is often related to built environments [25,28,29]. Further, sky visibility was previously found to be positively associated with naturalness as well as preference ratings [32]. The positive association between sky pixel ratio and view quality ratings might also be attributable to the positive effects of brightness. Relatedly, Beute and De Kort [75] found clear explicit preferences for natural, sunny, and bright environments. Future studies should thus include sky view (including subjective and objective ratings) as an important window view element and could investigate mediation via brightness. 

Finally, the number of images sent in by participants was positively associated with the rating of view quality in our study. A possible explanation for this effect could be that participants who like their window views more might have been more motivated to send in more photographs, whereas those who like their views less might only have sent in images of a subset of their windows.

All in all, the green pixel ratio is a highly important predictor for natural and (absence thereof) for man-made elements in the window view. Elements that were positively associated with view quality tend to be positively associated with natural views (saturation, sky pixel ratio, fractal dimension) and are at the same time rather negatively associated with the perceived percentage of man-made elements (sky pixel ratio). A possible explanation for the association of naturalness and view quality (proximally reflecting ‘preference’) is offered by the Stress Reduction Theory (SRT; [76,77]), which proposes that due to human evolutionary history and our close ties to the natural world, natural environments elicit a positive affective response and aesthetic appraisal (and thereby contribute to restorative effects). It is, however, important to note that all of the final models explained less than 25% of variance (22.92% for naturalness, 16.13% for man-made elements, 14.16% for view quality), thus, other features of window views (e.g., the recognition of specific natural elements or higher-level visual features, cf. [27,78,79]), dynamic features [80,81], or aspects that are not related to window views at all probably play an important role as well.

### 4.3. Predicting Mental Health Outcomes with Window View Parameters

The subjective brightness of rooms was negatively associated with state negative affect, i.e., brighter apartments may be more beneficial for affect, which is in line with previous findings on objective brightness [17,82,83,84] and with the SRT [76,77]. The finding is also broadly consistent with self-reported inadequate residential lighting relating to higher odds (*OR* = 1.4) of reporting a depression diagnosis [85] and with the literature on the role of light in the treatment of depression (for a meta-analysis see [86]). We previously concluded that the objective low-level visual feature brightness is not a reliable indicator of the actual brightness of a window in our study due to different contrasts depending on specific camera settings and different lighting conditions in the rooms of the participants. Also, the brightness of the window itself does not necessarily reflect the brightness within a room. However, given the potential importance of brightness in mitigating negative affect, future studies might want to include an alternative objective measure of brightness. This could either be completed with photographs that are taken in a more standardized manner (i.e., with the same camera and camera settings and during the same time of the day) or by using a luxmeter (see [17]). None of the other subjective window view ratings and none of the low-level visual features were associated with negative affect when testing all variables in a stepwise manner.

For impulsive decision-making in a delay discounting task, we found that the more time participants reportedly spent indoors, the lower their impulsive decision-making was. There are numerous potential explanations for this finding, e.g., participants who spend more time indoors might be generally more risk-averse and less impulsive than those who spend more time outdoors, or alternatively, staying indoors preserves inhibitory resources that outside would be in higher demand. However, this is pure speculation since we did not measure risk aversion or similar factors. Since the question on how much time participants spent indoors referred to the time before the COVID-19 restrictions, this finding cannot be attributed to better adherence to state regulations by less impulsive participants. The effect of time spent indoors during the COVID-19 restrictions did not reach significance. 

Further, we found that window views with a higher color saturation were negatively associated with impulsive decision-making. While the association between saturation and nature was inconsistent in previous studies [25,27,28,29], there was a significant positive correlation between the saturation and perceived percentage of nature in our sample as well as a significant negative correlation between the saturation and perceived percentage of man-made elements (saturation was also included in the final stepwise model predicting the perceived percentage of nature based on AIC, but the effect was not significant). This indicates that more natural views—which have a higher saturation in our sample—are associated with reduced impulsive decision-making. Similarly, earlier research also showed that, for young girls, a more natural view from home was associated with better performance in tests of concentration, impulse inhibition, and the delay of gratification [87]. 

However, we found contradictory results when it comes to overall edge density and the perceived percentage of nature. Even though both were positively associated with a more natural view, they were associated with increased impulsive decision-making. At first glance, this seems to contradict previous studies [19,35] in which (objective and subjective) nature experience was associated with reduced impulsive decision-making, although these studies did not examine window views specifically. However, the results may align with the Attention Restoration Theory (ART; [88,89]), according to which natural environments have inherent characteristics such as ‘soft fascination’ or a feeling of ‘being away’ which counteract the cognitive and attentional effort that come along with urban daily life challenges. Perhaps individuals with more depleted self-control—in a compensatory effort—unconsciously or consciously pay more attention to natural elements, which could explain the positive association between the perceived percentage of nature and higher levels of impulsive decision making. In addition, such individuals could per se select housing conditions that are less cognitively demanding (i.e., more natural). 

In summary, results regarding delay discounting were inconclusive: while saturation—which was associated with naturalness in our sample—was related to less impulsive decision-making, the effect of overall edge density and the perceived percentage of nature went in the opposite direction. However, the existing evidence mostly suggests that viewing natural scenes (compared to urban scenes) is associated with reduced impulsive decision-making in delay discounting tasks ([19,35], but see also [36]). It has to be noted that those studies compared viewing ‘purely’ natural vs. urban scenes, while our study used photographs of urban window views with varying amounts of natural elements (i.e., mixed environments), for which effects on delay discounting might be less pronounced. 

Faber Taylor et al. [87] found a positive effect of natural views from home on a combined measure of self-discipline. Similar to our study, participants rated the naturalness of their window views in an inner-city environment. However, previous studies suggest that self-discipline and impulsive decision-making are different constructs [90,91]. Further, the positive effect of natural views from home on self-discipline was only found for young girls and the authors suggest that for young boys, who tend to spend more time outside and away from home [92], distant nature might be equally important. This points to the importance of nature accessibility, which also emerged in a study by Repke et al. [19]: While both nature exposure from home (including window views) and nature accessibility predicted reduced scores on depression, anxiety, and stress scales as well as improved health and wellbeing, only nature accessibility predicted reduced impulsive decision-making. In the present study, a varying degree or lack of accessibility to the nature visible from our participants’ window views might explain inconsistencies in the effects of natural features on impulsive decision-making. 

Moreover, Repke et al. [19] propose a model according to which nature reduces impulsive decision-making via expanding space perception. They found that viewing images of natural compared to built scenes increased space perception and that an expanded space perception predicted reduced impulsive decision-making. Thus, the ability of natural scenes to induce a sense of vast scale might be critical for effects on impulsive decision-making. Window views—especially of mixed environments like in the present study—might not be able to prompt the same sense of vast scale as a ‘purely’ natural scene, which is likely to present more expansive landscapes. Small windows might also prevent views from being perceived as expansive. While we did not measure perceived openness or space perception of window views in our study, we asked participants to estimate how far they can see from their windows (‘long-distance view’). The perceived long-distance view did not turn out to be a significant predictor in the model predicting delay discounting (or any other model in the present study), but correlative patterns suggest that the perceived long-distance view is positively associated with view quality, subjective brightness, and with subjective and objective measures of sky. Future research might thus investigate whether and how much brightness or sky visibility influence how expansive a natural scene is perceived and by that, contribute to the effect of nature on delay discounting via expanded space perception.

Of note, we generally expect the effects of window views on cognition to be more short-lived than effects on wellbeing and suggest testing these effects more directly, e.g., in an experimental design. To explain a bit further, it is possible that the overall atmosphere at home, created among other factors by window views, has a generic effect on the mood state of a person. This is underlined by the fact that our findings regarding state negative affect were similar to those regarding trait negative affect (see Appendix D for details). However, for cognitive performance, the specific context while performing the task (sitting at or looking out of the window while performing the task)—which is unknown in the present study—may be of relevance. 

Further research is necessary to corroborate our findings, resolve inconsistencies (especially regarding delay discounting) and perform more targeted analyses on the association between specific window view features and mental health. For both mental health outcomes, the final models explained less than 20% of the variance, highlighting the important role of factors besides the window view on negative affect and impulsive decision-making. Other potentially relevant factors should thus be explored in future research, e.g., current mood states, circadian rhythms (time of assessment), and environmental context factors (where and under which conditions the task is performed).

### 4.4. Limitations

There are certain aspects of window views that cannot be captured in a photograph, e.g., ephemeral features like moving clouds or a flock of birds passing by. We asked participants to not include people in their photographs, however, the amount of people visible from the window might influence the view quality positively (e.g., by offering a connection to one’s social environment) or negatively (e.g., if one feels watched within their home). To approximate how these factors might affect the perception of window views, we asked participants for subjective ratings of their window views (including a rating of view quality). Still, non-visual features like noise or air quality can affect perceived naturalness as well as mental health (e.g., [93,94]) but were not examined in the present study. 

Another point of criticism concerns the focus of the research. The goal of improving window views to achieve beneficial effects on mental health might be viewed as undesirable, as it might motivate people to stay indoors even more. On the other hand, a beautiful and natural window view might be an incentive to go outside, which warrants further investigation. Nonetheless, given that people already spent a lot of their time indoors [95] and that especially elderly individuals might be bound to their homes due to reduced mobility, it is important to make the home environment, including window views, as health-promoting as possible in order to enhance wellbeing and mental health in these populations.

There are several methodological aspects concerning the window view photos to be considered. While the photographs of real window views including objects in front of the view (e.g., curtains or plants on windowsills) assured ecological validity, it came with several challenges. Since participants took the photographs themselves, they were taken under less-controlled conditions than, e.g., naturalistic window view photos that are taken by the experimenter [17,79]. Participants in the present study used different (phone) cameras and camera settings and their indoor lighting conditions varied, which lead to different contrasts between (the objects) indoors and the window views. The sanity checks of all images confirmed that the objective brightness values did not reflect the actual brightness of the view and were thus unusable. Given that subjective brightness turned out to be the only significant predictor for negative affect in our study, this limitation should be addressed in future studies by either taking all photographs with the same camera (settings) and under more controlled conditions, or by exploring alternative objective measures for brightness, e.g., using a luxmeter (confer [17]). Further, we did not ask participants to measure the actual height and width of their windows even though a smaller window could have a different effect than a bigger window with the same view. Again, this is especially true for features like brightness. Thus, we suggest window size as an important control variable for future studies. 

We focused on low-level visual features that were examined in multiple previous studies on image analyses in the context of perceived naturalness [25,27,28,29]. Of course, other features than those covered in the current study could be examined, e.g., spectral slope or symmetry [29]. Symmetry especially is an important part of human visual perception that often occurs automatically (i.e., without requiring attentional efforts), is crucial for perceiving three-dimensional objects as such, and affects preference [96,97]. While we assessed fractal dimension, which encompasses aspects of symmetry (scale symmetry/invariance), existing research suggests that vertical mirror symmetry in particular is easily detected and associated with preference (for a review see [96]). Relatedly, we found a positive association between the fractal dimension and ratings of view quality. However, future studies should add objective and subjective measures of mirror symmetry, especially when investigating ratings of view quality or preference. 

Another topic of limitation is the statistical and data aggregation approach employed in the present study. We used a stepwise regression approach which is often criticized because of its lack of theoretical considerations and other statistical problems [65]. However, it can be used for exploratory model building. Since no previous studies to our knowledge investigated how an exhaustive selection of low-level visual features vs. subjective ratings of window views affect mental health outcomes, we nonetheless decided to utilize this atheoretical approach. Further, we used a bidirectional stepwise selection, which removes some of the issues of a forward stepwise regression, e.g., the exclusion of variables involved in suppressor effects (for more details see [65]). Lasso regression offers an alternative approach for variable selection. We have reported lasso regression models for all outcomes (perceived naturalness, perceived man-made elements, view quality, state negative affect, and delay discounting) and compared them to the stepwise regression models in Appendix E. Most of the predictors that turned out to be significant in stepwise regression models were also included in lasso regression models (with the exception of delay discounting, supporting our idea that performance context needs to be controlled in future studies on window views and cognition). However, only stepwise regression allowed the block-wise inclusion of variable subsets and was thus deemed more appropriate for our research question. Another issue of stepwise regression is that of selective inference, meaning that the preselection of variables can lead to an exaggeration of the strength of effects [98]. For that reason, our approach is mainly intended to generate first insights and hypotheses that can inform future research on more targeted analyses of window view features and mental health. 

Furthermore, a larger sample size might have been better to detect potential effects. No power calculations were performed to determine sample size since we chose an exploratory approach and had no comparable studies on window views during the time of recruitment (May to June 2020) that could have informed power analysis. Yet, previous studies on associations between low-level visual features and naturalness [25,28,29] used sample sizes between 50 and 100 participants, therefore we aimed at a sample size of *N* = 100 to assess whether correlational results could be replicated for ecologically valid window view photographs. More recent studies on window views and mental health [23,24] used much larger sample sizes. While this was not feasible for our study, future research should perform a priori power calculations to ensure an adequate sample size.

A final area of limitations considers the control of confounders. Although we controlled for the time participants spent indoors, we do not know how long they were exposed to specific window views. We tried to identify participants’ most-used windows in two ways: For each image that was sent in, participants indicated how much time they spent in the respective room on a daily basis. In addition, they named the rooms with the windows they perceived mostly in the questionnaire. However, the reports were inconsistent for 45 of 109 (41.3%) participants and thus overall deemed unreliable. Studies in more controlled settings (e.g., schools, clinical environments, or senior residencies) might have better ways of identifying the exposure to specific windows and could use this to investigate a dose-dependent effect of window views on (mental) health.

## 5. Conclusions

This is the first study that performed an in-depth analysis of objective low-level features and the subjective perceptions of window views in participant homes under realistic conditions. We found that the amount of green in the view was most important for predicting the perceived percentage of nature and that low-level visual features associated with natural environments (higher fractal dimension and saturation, lower straight edge density) as well as the amount of visible sky contributed to the perceived quality of the window view. With regard to mental health, only a higher perceived brightness of rooms was beneficially related to affect, while effects on impulsive decision-making in a delay discounting task were less conclusive, indicating the need for future research to explore these associations in more controlled experiments. 

Considering the implementation of our findings, architects or city planners may aim to enhance the brightness within apartments and neighborhoods (e.g., with larger windows and less dense building complexes) to mitigate negative affect in the population. Further, it is crucial to maximize green space (also particularly from a climate and public health point of view), which would also increase the number of fractals (often found in trees or plants like ferns) in window views. This could enhance perceived naturalness and view quality, which might indirectly improve wellbeing. While further research is necessary to corroborate this, a salutary window view promises to be a viable way to improve mental health outcomes at the societal level.

## Figures and Tables

**Table 1:** Sociodemographic characteristics of the sample.

Sample Characteristics	*n* ^1^	%	Mean	*SD*
**Age (in years)**			26.8	7.99
**Sex**				
Male	28	25.45		
Female	81	73.64		
Diverse	1	0.01		
**Marital Status**				
Single	58	52.73		
Married	10	9.09		
Living in a relationship	42	38.18		
**Net Income**				
Below EUR 1250/month	39	35.45		
EUR 1250–1749/month	16	14.55		
EUR 1750–2249/month	11	10.00		
EUR 2250–2999/month	24	21.82		
EUR 3000–3999/month	10	9.09		
EUR 4000–4999/month	5	4.55		
Over EUR 5000/month	5	4.55		
**Education**				
High-school diploma	5	4.55		
Advanced technical certificate	6	5.45		
Higher education entrance qualification (e.g., Abitur)	99	90.00		
**Professional Qualification**				
Completed studies at university or college	42	38.18		
Currently studying at a university or college	4	3.64		
Completed vocational training	13	11.71		
Currently in vocational training	2	1.82		
No professional qualification	1	0.91		
**Current place of residence**				
City (population > 100,000)	96	87.27		
Town (population > 10,000)	7	6.35		
Rural environment (population < 10,000)	6	5.45		
**Living space in square meters**			81.32	88.54
**Number of rooms (without kitchen and bathroom)**			3.08	4.36
**Number of photographs that were sent in**			5.58	4.01

^1^ *N* = 110.

**Table 2:** Exemplary windows views and their low-level visual features ^1^.

Type	Window 1	Window 2
1. Hue	1.74	1.54
2. *SD* Hue	1.05	0.82
3. Saturation	0.25	0.21
4. *SD* Saturation	0.16	0.17
5. Brightness	0.38	0.41
6. *SD* Brightness	0.26	0.29
7. Green Pixel Ratio	0.23	0.13
8. Blue Pixel Ratio	0.08	0.01
9. Sky Pixel Ratio	0.11	0.13
10. Overall Edge Density	0.11	0.10
11. Straight Edge Density	0.05	0.04
12. Non-Straight Edge Density	0.08	0.07
13. Entropy	7.55	7.26
14. Fractal Dimension	1.79	1.81
Image	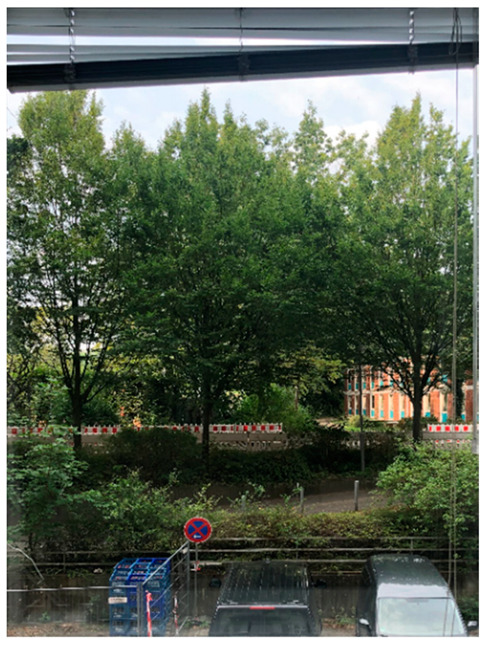	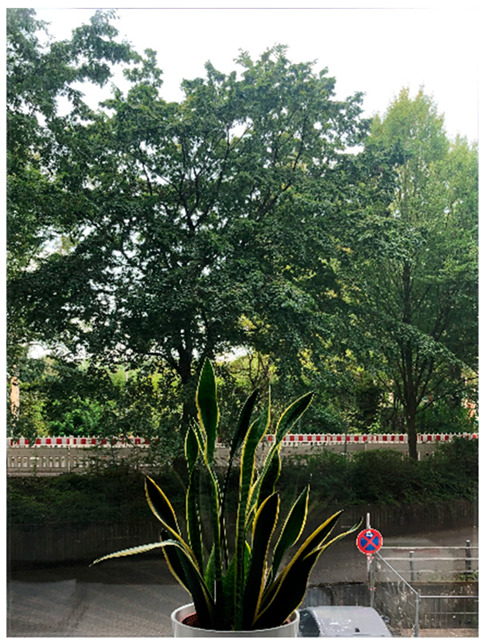

^1^ If not stated otherwise, the depicted values are average values. Features that are preceded by ‘*SD*’ show the standard deviation of the respective low-level visual feature.

**Table 3:** Stepwise regression procedure to predict perceived percentage of nature, perceived percentage of man-made elements, and view quality with low-level visual features ^1^.

Stepwise Included Predictors	Criteria
Hue	Perceived Percentage of Nature
*SD* of Hue	Perceived Percentage of Man-Made Elements
Saturation	Rating of View Quality
*SD* of Saturation	
Green Pixel Ratio	
Blue Pixel Ratio	
Sky Pixel Ratio	
Overall Edge Density	
Straight Edge Density	
Non-Straight Edge Density	
Entropy	
Fractal Dimension	

^1^ *SD* = Standard deviation.

**Table 4:** Hierarchical stepwise regression procedure to predict perceived percentage of nature, perceived percentage of man-made elements, and view quality with low-level visual features and control variables.

Block 1: Fixed Predictors (All Included)	Block 2: Stepwise Included Predictors	Criteria
Low-Level Features that were established in the previous stepwise regression (see Table 3)	Age Sex Income Time Spent at Home Before the COVID-19 Restrictions Time Spent at Home During the COVID-19 Restrictions Size of Apartment Image Count	Perceived Percentage of Nature Perceived Percentage of Man-Made Elements Rating of View Quality

**Table 5:** Hierarchical stepwise regression procedure to predict negative affect (STADI) and impulsive decision-making (delay discounting) with control variables, low-level visual features, and subjective window view ratings ^1^.

Block 1: Fixed Predictors (All Included)	Block 2: Stepwise Included Predictors		Criteria
	Subjective Ratings of Window Views	Low-Level Visual Features	
Age Sex Income Time Spent at Home Before the COVID-19 Restrictions Time Spent at Home During the COVID-19 Restrictions Size of Apartment Image Count	Perceived Percentage of Nature Perceived Percentage of Man-Made Elements Rating of View Quality Rating of Brightness in the Room Rating of Visibility of Green Rating of Visibility of Vegetation Rating of Long- Distance View	Hue *SD* of Hue Saturation *SD* of Saturation *SD* of Saturation Green Pixel Ratio Blue Pixel Ratio Sky Pixel Ratio Overall Edge Density Straight Edge Density Non-Straight Edge Density Entropy Fractal Dimension	State Negative Affect (STADI) Impulsive Decision-Making (Delay Discounting)

^1^ *SD* = Standard deviation.

**Table 6:** Multiple linear regression of perceived percentage of nature on low-level visual features.

Variable	*B*	*SE* ^1^	Β	*t*	*p*	Adjusted *R*^2^
**Final Model**						22.92% ***
Constant	10.78 *	5.05		2.14	0.03 *	
Green Pixel Ratio	56.28 **	18.20	0.35	3.09	0.003 **	
Saturation	42.153	25.81	0.19	1.63	0.11	

^1^ *SE* = Standard error. * *p* < 0.05. ** *p* < 0.01. *** *p* < 0.001. *N* = 109.

**Table 7:** Multiple linear regression of perceived percentage of man-made elements on low-level visual features.

Variable	*B*	*SE* ^1^	Β	*t*	*p*	Adjusted *R*^2^
**Final Model**						16.13% ***
Constant	75.40 ***	10.35		7.29	<0.001 ***	
Green Pixel Ratio	−87.08 ***	18.91	−0.52	−4.61	<0.001 ***	
Sky Pixel Ratio	−34.87 **	12.79	−0.26	−2.73	0.01 *	
*SD* ^2^ of Hue	−14.00 *	6.95	−0.22	−2.01	0.046 *	

^1^ *SE* = Standard error. ^2^ *SD* = Standard deviation. * *p* < 0.05. ** *p* < 0.01. *** *p* < 0.001. *N* = 109.

**Table 8:** Multiple linear regressions of view quality ratings on low-level visual features ^1^.

Variable	*B*	*SE* ^2^	Β	*t*	*p*	Adjusted *R*^2^
**Model 1**						11.15% **
Constant	−107.73	72.74		−1.48	0.14	
Saturation	63.64 *	28.12	0.22	2.26	0.03 *	
Sky Pixel Ratio	44.65 **	16.19	0.27	2.76	0.007 **	
Fractal Dimension	92.26 *	42.66	0.24	2.16	0.03 *	
SED ^3^	−118.13	63.60	−0.18	−1.86	0.07	
**Model 2**						14.16% ***
Constant	−128.23	72.13		−1.78	0.08	
Saturation	62.17 *	27.65	0.22	2.25	0.03 *	
Sky Pixel Ratio	45.70 **	15.92	0.28	2.87	0.005 **	
Fractal Dimension	100.50 *	42.11	0.26	2.39	0.02 *	
SED ^3^	−126.20 *	62.63	−0.19	-2.02	0.046 *	
Image Count	1.22 *	0.57	0.19	2.16	0.03 *	

^1^ Model 1 shows the results of the automated bidirectional stepwise linear regression with all low-level visual features as possible predictors. For model 2, all predictors identified as significant in stepwise regression model 1 were included and in addition an automated bidirectional stepwise linear regression was used to identify relevant control variables. ^2^ *SE* = Standard error. ^3^ SED = Straight edge density. * *p* < 0.05. ** *p* < 0.01. *** *p* < 0.001. *N* = 109.

**Table 9:** Multiple linear regression of state negative affect on window parameters.

Variable	*B*	*SE* ^1^	Β	*t*	*P*	Adjusted *R*^2^
**Final Model**						15.45 **
Constant	−3.16	28.12		−0.11	0.91	
Age	−0.11	0.12	−0.09	−0.96	0.34	
Sex	0.13	2.14	0.01	0.06	0.95	
Income	−0.37	0.53	−0.07	−0.70	0.49	
Living Space in qm	−0.01	0.01	−0.08	−0.77	0.44	
Time spent at Home	0.28	0.19	0.15	1.49	0.14	
Time spent at Home (during COVID-19) ^2^	0.13	0.31	0.04	0.42	0.68	
Image Count	−0.27	0.25	−0.11	−1.09	0.28	
Brightness Rating	−0.12	0.04	−0.29	−3.15	0.002 **	
Entropy	6.31	3.64	0.17	1.73	0.09	
SED ^3^	41.76	25.63	0.16	1.63	0.11	

^1^ *SE* = Standard error. ^2^ Time spent at Home (during COVID-19) refers to the time spent at home during the COVID-19 restrictions. ^3^ SED = Straight edge density. ** *p* < 0.01. Two observations were excluded due to missing data, such that *N* = 107.

**Table 10:** Multiple linear regression of delay discounting (AUC ^1^ value) on window parameters.

Variable	*B*	*SE* ^2^	β	*t*	*p*	Adjusted *R*^2^
**Final Model**						12.20 *
Constant	−96.31	68.53		−1.41	0.16	
Age	0.30	0.29	0.10	1.01	0.32	
Sex	4.64	5.29	0.08	0.88	0.38	
Income	−0.59	1.32	−0.04	−0.45	0.66	
Living Space in square meters	−0.007	0.03	−0.03	−0.26	0.80	
Time spent at Home	0.95	0.47	0.22	2.05	0.04 *	
Time spent at Home (during COVID-19) ^3^	0.82	0.78	0.12	1.06	0.29	
Image Count	−0.07	0.60	−0.01	−0.11	0.91	
Saturation	79.42	30.91	0.29	2.57	0.01 *	
Overall Edge Density	−241.32	114.21	−0.25	−2.11	0.04 *	
Fractal Dimension	64.32	42.29	0.18	1.52	0.13	
Perceived Percentage of Nature	−0.38	0.16	−0.31	−2.34	0.02 *	
Rating of Visibility of Green	0.17	0.11	0.20	1.56	0.12	

^1^ AUC = Area under the Curve. A higher AUC value of the delay discounting task represents less discounting by delay, i.e., less impulsive decision-making. ^2^ *SE* = Standard error. ^3^ Time spent at Home (during COVID-19) refers to the time spent at home during the COVID-19 restrictions. * *p* < 0.05. One observation was excluded due to missing data, such that *N* = 109.

**Figure A1:**
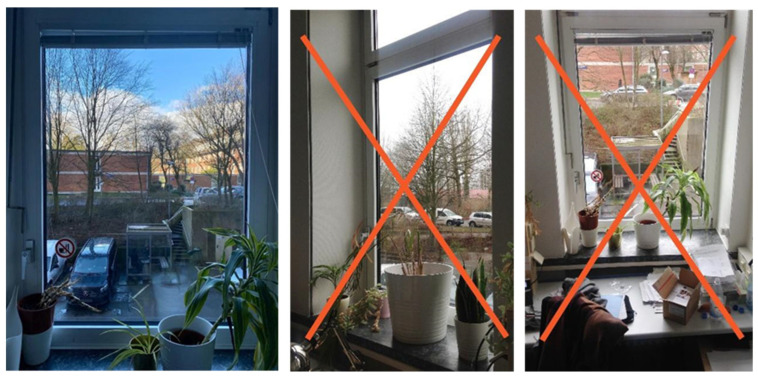
Example window view photographs including window frames.

**Figure A2:**
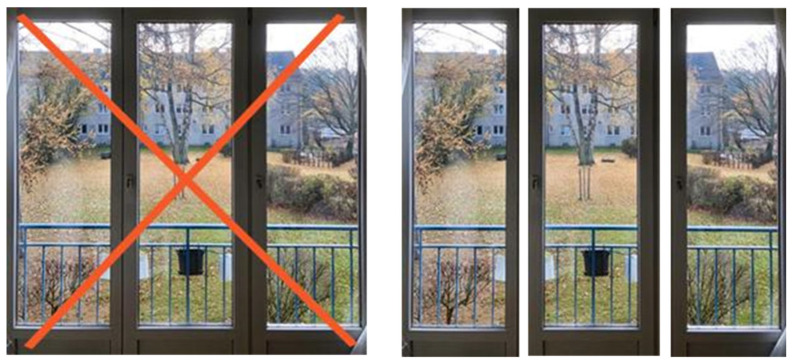
Example window view photographs of several windows next to each other.

**Figure A3:**
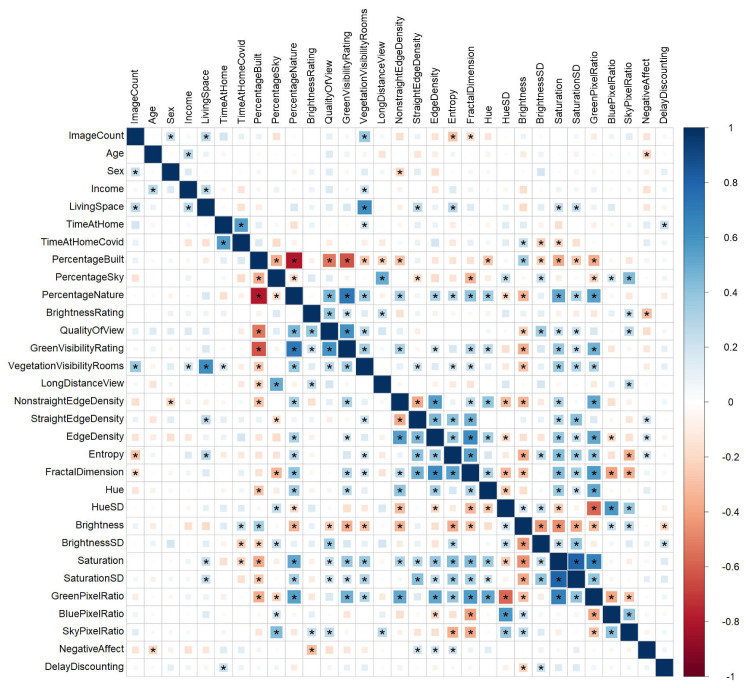
Spearman correlations between all variables of interest. PercentageNature = perceived percentage of nature in the window view; PercentageBuilt = perceived percentage of man-made elements in the window view; PercentageSky = perceived percentage of sky in the window view; QualityOfView = rating of overall view quality; BrightnessRating = rating of brightness in the room; GreenVisbilityRating = rating of visibility of green in the window view; LongDistanceView = estimation how far one can see out of the windows; NegativeAffect = state negative affect. Low-level visual feature values were averaged across all window views of each participant. *SD* = Standard Deviation. * *p* < 0.05.

## Data Availability

The data presented in this study are openly available in the Open Science Framework (OSF) at doi.org/10.17605/OSF.IO/6X8FY. The original window view photographs are not publicly available due to participant privacy.

## Appendix A. Instructions for Taking the Window View Photos (Translated from German)

Dear study participant,

Thank you for taking part in our “Window on the World” study. After completing the online questionnaires, we would like to ask you to take photos of the windows of your current home. We use a website for this purpose, which you can use to send us the photos. Please be assured that we will handle your data in accordance with data protection regulations. In order to be able to evaluate the photos, it is important that they meet certain requirements. Therefore, please follow these instructions for taking the photos.

1. After completing the questionnaires, please send us an email with your study ID and telephone number. We will then send you a text message (SMS) with a link to your cell phone.
2. If you click on this link, you will be redirected to a website. We have created this so that you can send us your window photos in a simple way.
3. You will first be asked which room you are currently in.
4. After entering your details, press the “Start camera” button and take a photo of the window. Please note the following:

- Please take the photo at a bright time of day (morning or midday).
- You do not need to clear your windowsills for the photo to be taken.
- If curtains, blinds, etc. almost constantly cover your window, please photograph the window with them.
- Please photograph frosted glass windows when they are closed.
- Please make sure that no people are visible in the photo.
- To take the photo, please stand up straight in front of the window and take the photo from your eye level (please hold the cell phone upwards or take the photo from the side).
- The window frames should be visible in the picture:

- If there are several windows next to each other (e.g., also a balcony door), please photograph each window individually. You can see sample photos here:

5. If the photo meets the above requirements, please click on “Use photo” (bottom right). The photo will then appear in the preview. Then, click on “Upload”. Be patient, after a short time you will receive a message that the photo has been successfully uploaded.
6. You will then be asked whether there are any other windows in the same room. Answer “Yes” or “No more windows”. As soon as you click on “No more windows”, you will be asked how long you usually spend in this room each day. Please enter the number of hours and minutes and click on “Next”.

Please take photos of all the windows in your home!

If you have any questions or difficulties using the application, we will be happy to help you: *[phone number and email address of the instructors]*

Thank you for your participation!

Your study team.

## Appendix B. Feature-by-Feature Report of Spearman Correlations Depicted in Figure 2

- We found that the **hue** was significantly negatively correlated with the perceived percentage of man-made elements (ρ = −0.26, *p* < 0.05) and significantly positively correlated with the perceived percentage of nature in the window view (ρ = 0.36, *p* < 0.05). These results are intuitive, given that in our study a higher hue reflects colors like yellow or green, while a lower hue reflects colors like blue or magenta. However, the hue did not correlate with the rating of view quality (ρ = 0.04, *p* = 0.70). While Menzel and Reese [29] found higher hue values for nature compared to urban images, most previous studies showed a negative correlation between hue and naturalness as well as hue and preference [25,27,28,73] and point out an increased preference for the color blue [27,73].
- The ***SD* of hue** was not significantly correlated to the perceived percentage of man-made elements (ρ = 0.05, *p* = 0.62), nor to those of nature (ρ = −0.18, *p* = 0.06), nor to the rating of view quality (ρ = 0.04, *p* = 0.89). In previous studies, the *SD* of hue was negatively associated with (perceived) naturalness [25,28,29]. While the correlation of the *SD* of hue and the percentage of nature in our study goes in the same direction, the effect was not significant.
- **Saturation** was significantly positively correlated with view quality (ρ = 0.22, *p* < 0.05) and the percentage of nature (ρ = 0.52, *p* < 0.05) and significantly negatively correlated with the percentage of man-made elements (ρ = −0.35, *p* < 0.05). Our results are in line with findings by Ibarra et al. [27] and Menzel and Reese [29] but contradict Kardan et al. [28]. Also, Berman et al. [25] found no univariate correlation between saturation and perceived naturalness.
- Similar results were found for the ***SD* of saturation**: A positive correlation with view quality (ρ = 0.20, *p* < 0.05) and perceived percentage of nature (ρ = 0.34, *p* < 0.05) as well as a negative correlation with perceived percentage of man-made elements (ρ = −0.24, *p* < 0.05). Previous studies also show a positive association between the *SD* of saturation and naturalness and preference [25,28,29].
- **Brightness** showed a significant positive correlation with the perceived percentage of man-made elements (ρ = 0.32, *p* < 0.05) and significant negative correlations with perceived percentage of nature (ρ = −0.33, *p* < 0.05) and view quality (ρ = −0.25, *p* < 0.05). Contrary to our results, previous studies found (small) positive associations of brightness with naturalness and preference [27] as well as with affective ratings [99], or no association at all [25]. While it is a possibility that brightness in our sample was sustained by built rather than by natural elements, sanity checks of the photographs did not clearly support this hypothesis. It seems that the low-level visual feature brightness is not a reliable estimate for the actual brightness of window views.
- The ***SD* of brightness** was significantly negatively correlated with the perceived percentage of man-made elements (ρ = −0.24, *p* < 0.05) and significantly positively correlated with view quality (ρ = 0.36, *p* < 0.05). There was no correlation between the *SD* of brightness and the perceived percentage of nature (ρ = 0.08, *p* = 0.39). Berman et al. [25] found no association between the *SD* of brightness and perceived naturalness, while Ibarra et al. [27] found a positive relationship. However, the above-mentioned flaws in the objective assessment of average brightness apply to the *SD* of brightness as well.
- As expected, the **green pixel ratio** was significantly negatively correlated with the perceived percentage of man-made elements (ρ = −0.34, *p* < 0.05) and significantly positively correlated with the perceived percentage of nature (ρ = 0.55, *p* < 0.05). However, there was no significant correlation between the green pixel ratio and view quality (ρ = 0.15, *p* = 0.11).
- **The blue pixel ratio** did not show significant correlations with view quality (ρ = 0.01, *p* = 0.96), the perceived percentage of nature (ρ = −0.07, *p* = 0.50) or the perceived percentage of man-made elements (ρ = −0.05, *p* = 0.63). Sanity checks of all window view photographs indicated that the blue pixel ratio in our sample did not reflect blue natural or man-made elements in the window view, but rather blue skies. Correspondingly, blue pixel ratio was positively correlated with sky pixel ratio (ρ = 0.42, *p* < 0.05) and the perceived percentage of sky in the window view (ρ = 0.23, *p* < 0.05).
- While **sky pixel ratio** was significantly positively correlated to view quality (ρ = 0.27, *p* < 0.05); it was not correlated with the perceived percentage of nature (ρ = −0.14, *p* = 0.14) or man-made elements (ρ = −0.14, *p* = 0.15) in the window view.
- **Entropy** was significantly positively correlated with the perceived percentage of nature (ρ = 0.27; *p* < 0.05), but not correlated with the perceived percentage of man-made elements (ρ = −0.14, *p* = 0.15) or view quality (ρ = 0.02, *p* = 0.85). This contradicts previous findings that found either no association [25] or a negative association [27] between entropy and naturalness.
- There was a significant positive correlation between **overall edge density** and the perceived percentage of nature (ρ = 0.32, *p* < 0.05), but no correlations with the perceived percentage of man-made elements (ρ = −0.12, *p* = 0.21) or view quality (ρ = −0.01, *p* = 0.93). Previous studies also show a positive association between overall edge density and naturalness [25,28,29].
- **Straight edge density** was not significantly correlated to view quality (ρ = −0.09, *p* = 0.37), the perceived percentage of nature (ρ = 0.07, *p* = 0.48) or the perceived percentage of man-made elements (ρ = 0.09, *p* = 0.35). In previous studies, however, straight edge density was negatively associated with naturalness [25,28,29].
- **Non-straight edge density** was significantly negatively correlated with the perceived percentage of man-made elements (ρ = −0.26, *p* < 0.05) and significantly positively correlated with the perceived percentage of nature (ρ = 0.33, *p* < 0.05), but not correlated with view quality (ρ = 0.11, *p* = 0.27). These findings are in line with previous studies [25,29].
- There was a correlation between **fractal dimension** and the perceived percentage of nature (ρ = 0.38, *p* < 0.05), but no correlation with the perceived percentage of man-made elements (ρ = −0.15, *p* = 0.12) or view quality (ρ = 0.03, *p* = 0.79). Findings by Menzel and Reese [29] also indicate a positive association between fractal dimension and naturalness, but Hagerhall et al. [100] suggest that medium values are preferred.

## Appendix D. Predicting Trait Negative Affect with Window View Parameters

For detailed results, see Table A1. The hierarchical stepwise linear regression analysis resulted in a model that included all control variables (due to forced entry in a first hierarchical block; however, none of them were significant) as well as subjective brightness, entropy, the perceived percentage of sky, hue, and non-straight edge density, which were selected in a stepwise manner from a second block (AIC_final model_ = 486.06 compared to AIC_control variables only_ = 491.33). The subjective rating of brightness in the rooms was significantly (*p* < 0.05) negatively associated with trait negative affect, i.e., a higher rating of brightness was associated with lower trait negative affect. On the other hand, entropy and hue were significantly (*p* < 0.05) positively associated with trait negative affect. All other predictors did not reach significance. In the final model, 14.44% of the variance in trait negative affect was explained, *F*(12, 94)= 2.49, *p* = 0.007.

**Table A1:** Multiple linear regression of trait negative affect on window parameters.

Variable	*B*	*SE* ^1^	β	*t*	*p*	Adjusted *R*²
**Final Model**						14.44 **
Constant	−3.80	28.91		−0.13	0.90	
Age	−0.14	0.12	−0.11	−1.18	0.24	
Sex	0.32	2.20	0.01	0.15	0.88	
Income	−0.75	0.53	−0.14	−1.42	0.16	
Living Space in qm	−0.01	0.01	−0.10	−1.01	0.32	
Time spent at Home	0.31	0.19	0.17	1.64	0.10	
Time spent at Home (during COVID-19) ^2^	−0.05	0.33	−0.02	−0.17	0.87	
Image Count	−0.44	0.25	−0.18	−1.73	0.09	
Brightness Rating	−0.09	0.04	−0.22	−2.32	0.02 *	
Entropy	7.01	3.51	0.20	2.00	0.05 *	
Perceived Percentage of Sky	0.13	0.07	0.18	1.81	0.07	
Hue	2.29	1.15	0.21	2.00	0.05 *	
NSED ^3^	−63.12	40.79	−0.16	−1.55	0.13	

^1^ *SE* = Standard error. ^2^ Time spent at Home (during COVID-19) refers to the time spent at home during the COVID-19 restrictions. ^3^ NSED = Non-straight edge density. Values are rounded up to two decimal places, significance levels refer to values before rounding. * *p* < 0.05. ** *p* < 0.01.

## Appendix E. Lasso Regression Analyses

Lasso regression offers an alternative variable selection method to stepwise regression. We performed lasso regression with RStudio for the six outcome variables: perceived percentage of nature, perceived percentage of man-made elements, view quality, state negative affect, and delay discounting, using the same predictors as in stepwise regression analyses (see Table 3, Table 4 and Table 5 in the main text). For all outcomes, we selected the tuning parameter λ that produces the lowest mean squared error by using 10-fold cross validation, utilizing the glmnet-package [101]. (Note that each run of cross validation produces a slightly differing λ value, resulting in slightly differing β coefficients.) Prior to performing lasso regression, all predictor variables were z-standardized. Below, we present the results and compare them to the stepwise regression results reported in the main text.

### Appendix E.1. Perceived Percentage of Nature in Window Views

**Table A2:** Lasso regression of perceived percentage of nature on standardized measures of low-level visual features.

Variable	β	*R*²
**Final Model**		20.87
Constant	30.35	
Saturation	1.67	
Green Pixel Ratio	5.00	

λ = 3.41. Predictors with coefficients equal to zero are not reported. *N* = 109.

Like in the stepwise regression approach, the green pixel ratio and saturation turned out to be relevant and positive predictors of the perceived percentage of nature, with the green pixel ratio having the largest β coefficient. The lasso model explained 20.87% of variance, whereas the stepwise regression model explained 22.92%.

### Appendix E.2. Perceived Percentage of Man-Made Elements in Window Views

**Table A3:** Lasso regression of perceived percentage of man-made elements on standardized measures of low-level visual features.

Variable	β	*R*²
**Final Model**		15.80
Constant	40.12	
Non-straight Edge Density	−1.69	
Saturation	−2.70	
*SD* ^1^ of Saturation	−0.04	
Green Pixel Ratio	−2.57	
Sky Pixel Ratio	−2.40	
Living Space in square meters	−0.49	

λ = 1.92. Predictors with coefficients equal to zero are not reported. ^1^ *SD* = Standard deviation. *N* = 109.

Both stepwise regression and lasso regression showed that green pixel ratio and sky pixel ratio are relevant predictors that are negatively associated with the perceived percentage of man-made elements. While only stepwise regression yielded the *SD* of hue as an important predictor, only lasso regression included non-straight edge density, saturation (mean and *SD*) and living space as predictors. The lasso model explained 15.80% of variance, which is slightly less than the stepwise regression model explained (16.13%).

### Appendix E.3. Ratings of View Quality

**Table A4:** Lasso regression of ratings of view quality on standardized measures of low-level visual features and control variables.

Variable	β	*R*²
**Final Model**		19.18
Constant	67.57	
Straight Edge Density	−2.49	
Fractal Dimension	4.10	
*SD* ^1^ of Hue	0.83	
Saturation	4.00	
Sky Pixel Ratio	5.00	
Age	2.65	
Sex	1.36	
Income	1.12	
Living Space in square meters	0.30	
Image Count	2.92	

λ = 1.27. Predictors with coefficients equal to zero are not reported. ^1^ *SD* = Standard deviation. *N* = 109.

Sky pixel ratio, fractal dimension, straight edge density, and image count were included as predictors in both the stepwise and lasso regression approach. Only the stepwise regression model included saturation, while only lasso regression yielded the *SD* of hue, age, sex, income, and living space as additional predictors. The lasso model explained 19.18% of variance, whereas the stepwise regression model explained 14.16%.

### Appendix E.4. State Negative Affect

**Table A5:** Lasso regression of ratings of state negative affect on standardized measures of window view features and control variables.

Variable	β	*R*²
**Final Model**		22.73
Constant	38.23	
Straight Edge Density	1.13	
Entropy	1.37	
Hue	0.51	
Perceived Percentage of Sky	0.18	
Brightness Rating	−2.13	
Green Visibility Rating	−0.74	
Age	−0.30	
Income	−0.41	
Living Space in qm	−0.32	
Time spent at Home	0.94	
Time spent at home (during COVID-19) ^1^	0.27	
Image Count	−0.36	

λ = 0.64. Predictors with coefficients equal to zero are not reported. ^1^ Time spent at Home (during COVID-19) refers to the time spent at home during the COVID-19 restrictions. *N* = 107.

In the block-wise stepwise regression approach, all control variables were included as fixed predictors and stepwise selection was used to identify the window view features (both subjective and objective) that best explained state negative affect. Since none of the control variables turned out to be significant, variable selection of control variables cannot be compared in a sensible manner. Regarding window view features, both stepwise regression and lasso regression included brightness ratings, entropy, and straight edge density as predictors. In addition, lasso regression included hue, the perceived percentage of sky, and green visibility rating. In both models, the brightness rating had the largest β coefficient out of all predictor variables. The lasso model explained 22.73% of variance, whereas the stepwise regression model explained 15.45%.

### Appendix E.5. Delay Discounting

**Table A6:** Lasso regression of delay discounting (AUC ^1^ value) on standardized measures of window view features and control variables.

Variable	β	*R*²
**Final Model**		5.86
Constant	54.44	
Saturation	0.87	
Time spent at Home	2.80	

λ = 3.58. Predictors with coefficients equal to zero are not reported. ^1^ AUC = Area under the Curve. A higher AUC value of the delay discounting task represents less discounting by delay, i.e., less impulsive decision-making. *N* = 108.

As described in the previous section, a block-wise stepwise regression approach was used, by which all control variables were included as fixed predictors and stepwise selection was used to identify window view features that best explained delay discounting. However, only the time spent at home (prior to the COVID-19 restrictions) turned out to be a significant predictor in the stepwise regression model and was also the only control variable included as a predictor in the lasso approach. The lasso regression yielded only one other predictor with a β coefficient unequal to zero: saturation. Saturation was also a significant predictor in the stepwise regression model, but other window view features (overall edge density, fractal dimension, perceived percentage of nature, and green visibility rating) were included here as well. The lasso model explained 5.86% of variance, which is less than the stepwise regression model explained (12.20%).
